# Pathogenicity Locus, Core Genome, and Accessory Gene Contributions to *Clostridium difficile* Virulence

**DOI:** 10.1128/mBio.00885-17

**Published:** 2017-08-08

**Authors:** Brittany B. Lewis, Rebecca A. Carter, Lilan Ling, Ingrid Leiner, Ying Taur, Mini Kamboj, Erik R. Dubberke, Joao Xavier, Eric G. Pamer

**Affiliations:** a Infectious Disease Service, Department of Medicine, Memorial Sloan Kettering Cancer Center, New York, New York, USA; b Center for Microbes, Inflammation and Cancer, Memorial Sloan Kettering Cancer Center, New York, New York, USA; c Weill Cornell Medical College, New York, New York, USA; d Infection Control, Department of Medicine, Memorial Sloan Kettering Cancer Center, New York, New York, USA; e Department of Medicine, Washington University School of Medicine, St. Louis, Missouri, USA; f Computational and Systems Biology Program, Memorial Sloan Kettering Cancer Center, New York, New York, USA; g Immunology Program, Sloan-Kettering Institute, New York, New York, USA; University of Oklahoma Health Sciences Center

**Keywords:** *Clostridium difficile*, accessory genome, bile salt, genome analysis, pathogenicity locus, toxin

## Abstract

*Clostridium difficile* is a spore-forming anaerobic bacterium that causes colitis in patients with disrupted colonic microbiota. While some individuals are asymptomatic *C. difficile* carriers, symptomatic disease ranges from mild diarrhea to potentially lethal toxic megacolon. The wide disease spectrum has been attributed to the infected host’s age, underlying diseases, immune status, and microbiome composition. However, strain-specific differences in *C. difficile* virulence have also been implicated in determining colitis severity. Because patients infected with *C. difficile* are unique in terms of medical history, microbiome composition, and immune competence, determining the relative contribution of *C. difficile* virulence to disease severity has been challenging, and conclusions regarding the virulence of specific strains have been inconsistent. To address this, we used a mouse model to test 33 clinical *C. difficile* strains isolated from patients with disease severities ranging from asymptomatic carriage to severe colitis, and we determined their relative *in vivo* virulence in genetically identical, antibiotic-pretreated mice. We found that murine infections with *C. difficile* clade 2 strains (including multilocus sequence type 1/ribotype 027) were associated with higher lethality and that *C. difficile* strains associated with greater human disease severity caused more severe disease in mice. While toxin production was not strongly correlated with *in vivo* colonic pathology, the ability of *C. difficile* strains to grow in the presence of secondary bile acids was associated with greater disease severity. Whole-genome sequencing and identification of core and accessory genes identified a subset of accessory genes that distinguish high-virulence from lower-virulence *C. difficile* strains.

## IMPORTANCE

*Clostridium difficile* is an important cause of hospital-associated intestinal infections, and recent years have seen an increase in the number and severity of cases in the United States. A patient’s antibiotic history, immune status, and medical comorbidities determine, in part, the severity of *C. difficile* infection. The relative virulence of different clinical *C. difficile* strains, although postulated to determine disease severity in patients, has been more difficult to consistently associate with mild versus severe colitis. We tested 33 distinct clinical *C. difficile* isolates for their ability to cause disease in genetically identical mice and found that *C. difficile* strains belonging to clade 2 were associated with higher mortality. Differences in survival were not attributed to differences in toxin production but likely resulted from the distinct gene content in the various clinical isolates.

## INTRODUCTION

*Clostridium difficile* is an opportunistic, Gram-positive pathogen and the etiologic agent of most cases of health care-associated intestinal infections (1). *C. difficile* exploits intestinal dysbiosis to induce profuse inflammatory diarrhea and colitis which, in extreme cases, can be lethal (2). Although *C. difficile* has been recognized as an important pathogen since the 1970s (3), the past 15 years have seen a rise in the number and severity of infections (4). These increases are partially attributed to the emergence of a strain referred to as multilocus sequence type 1 (MLST 1) and ribotype 027 (R027) (5). Today, *C. difficile* is recognized as an urgent threat to the health care system, with an estimated half million infections per year in the United States and at least 3.8 billion dollars in excess medical costs (1, 2).

*C. difficile* produces two potent cytotoxins, toxin A and toxin B, which disrupt the actin cytoskeleton of intestinal epithelial cells (6). The toxins compromise the epithelial barrier, which leads to the translocation of commensal bacteria and influx of inflammatory cells. However, not all patients exposed to *C. difficile* develop colitis; some patients become asymptomatically colonized or have only self-limited episodes of diarrhea. For example, one study in a long-term-care facility found that 51% of residents were colonized with *C. difficile* despite a lack of apparent clinical illness (7). More recently, a prospective study of patients receiving bone marrow transplants reported an asymptomatic carriage rate of 27% (8). It is not clear what distinguishes asymptomatic patients from those who experience clinical infection, as the colonizing strains are frequently toxin producers and members of epidemic lineages.

Previous work has highlighted the importance of a healthy gut microbiota and intact immune system to prevent and recover from *C. difficile* challenge (9–15). In contrast, it has been more difficult to identify *C. difficile* strains that are consistently associated with severe disease versus mild diarrhea or asymptomatic colonization. Patients with *C. difficile* infection have a range of other diseases (comorbidities), variable states of immune function, and generally have received diverse combinations of microbiota-disrupting antibiotics (8, 16–20). These complex clinical variables make it difficult to distinguish the role of strain differences in the severity of colitis.

To circumvent these complexities, we have used a mouse model of *C. difficile* infection and disease progression. Mice share with humans many of the key aspects of *C. difficile*-associated disease, including antibiotic-induced susceptibility (9, 11, 21, 22), inflammatory cell recruitment to the colonic mucosa (13), and chronic carriage (23). In contrast to patient populations, however, our experiments have used genetically identical mice with similar microbiota compositions that receive identical antibiotic pretreatment prior to infection with precisely quantified inocula of *C. difficile* spores.

We obtained 33 phylogenetically diverse *C. difficile* isolates from patients hospitalized at Memorial Sloan Kettering Cancer Center (MSKCC) and Barnes-Jewish Hospital, infected mice with these *C. difficile* isolates, and monitored them for signs of symptomatic disease. *In vitro* assays combined with *in silico* analyses of whole-genome sequences revealed bacterial phenotypes and genetic variants associated with virulence. We found that that clinical isolates belonging to *C. difficile*’s clade 2 (particularly, the MLST 1/R027 strain) resulted in higher lethality in mice but that these differences in virulence were not attributable to the amount of toxin production or differences in toxin gene sequence. Strains with highly similar core genome sequences were found to cause a range of disease severity in the host, suggesting that *in vivo* pathology is, at least in part, attributable to differences in noncore, accessory gene representation.

## RESULTS

### Clinical isolates produce a range of disease severity and mortality.

We obtained a diverse array of *C. difficile* clinical isolates from patients hospitalized at MSKCC (*n* = 21) and Washington University’s (WU) academic medical center affiliate, Barnes-Jewish Hospital (BJH; *n* = 12) and classified the isolates by using MLST (24, 25). Of the 33 isolates examined, 24 belonged to *C. difficile* clade 1 (with MLSTs 2 and 42 most prevalent), 6 were classified as clade 2 (5 of which were MLST 1/ R027), 1 was classified as clade 4 (MLST 39), and 2 were classified as clade 5 (both MLST 11).

Clinical isolates obtained from WU/BJH were assigned human virulence scores as described previously (26). Briefly, severe disease was recorded if the patient had clinically significant diarrhea with a white blood cell count of ≥15,000 cells/mm^3^ and/or a serum creatinine level that was ≥1.5 times the premorbid level at the time of *C. difficile* diagnosis. Clinical isolates obtained from MSKCC could not be similarly scored because samples were collected from patients receiving bone marrow transplants and cancer chemotherapy, and their disease process results in low white blood cell counts that do not rise after *C. difficile* infection. MSKCC patients were classified as “*C. difficile* severe” if they had serum creatinine levels that were ≥1.5 times the premorbid level and had confirmed colitis, as evidenced by imaging.

**FIG 1:**
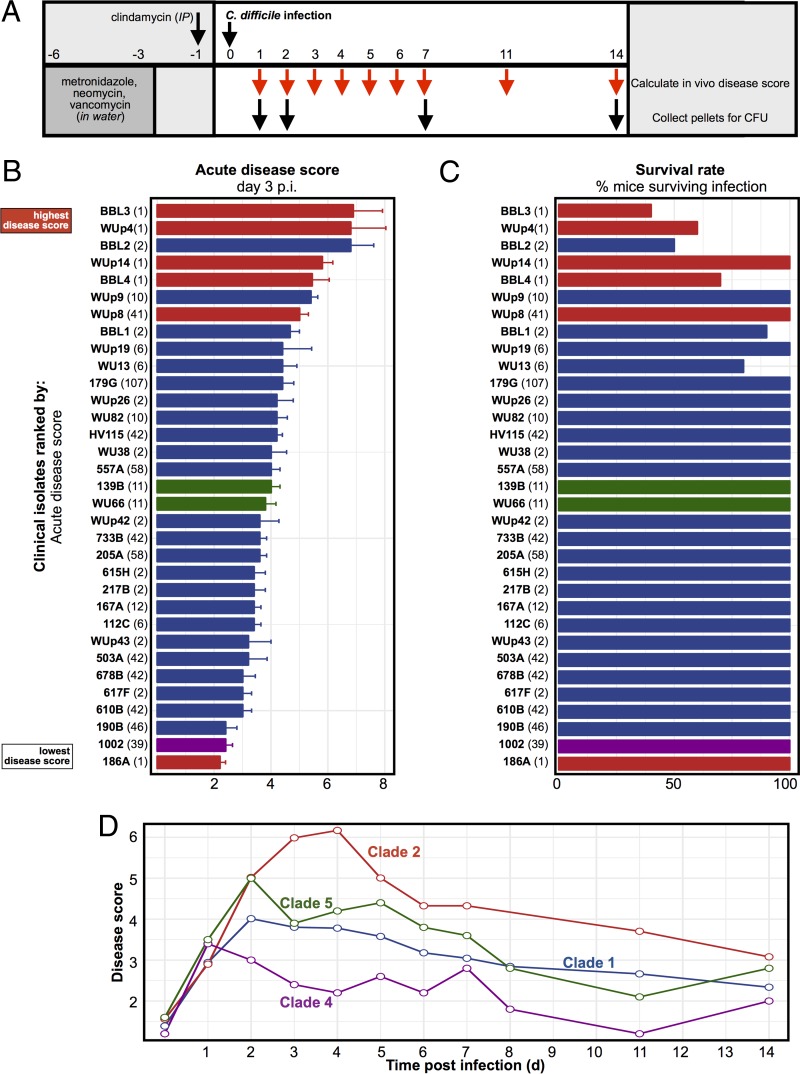
*In vivo* disease burden of *C. difficile* clinical isolates in the mouse model. (A) Summary of the experimental protocol. (B) Average acute disease scores taken 3 days postinfection for each of the clinical isolates (*n* = 5 to 10 mice per isolate). (C) Survival rates in the 2 weeks following infection. Clinical isolates are labeled by their unique strain identifier (in boldface) followed by their MLST type (in parentheses). Error bars indicate standard errors. (D) Disease scores following infection. Disease scores for each clinical isolate were averaged by clade.

Wild-type C57BL/6 mice were rendered susceptible to *C. difficile* infection by treatment with a cocktail of several antibiotics (see Materials and Methods) (Fig. 1A). Twenty-four hours after antibiotic cessation (designated day 0), the mice were orally gavaged with 100 spores from specific *C. difficile* clinical isolates and then monitored for the next 2 weeks for measures of disease burden and *C. difficile* colonization. Maximum weight loss and peak disease occurred between days 2 and 4 postinfection. Each clinical isolate was therefore assigned an “acute disease score,” which corresponded with the average disease score on day 3 after infection (Fig. 1B).

Most mice recovered from the infection (Fig. 1C). For example, only 2/24 strains from clade 1 and none of the three strains belonging to clades 4 and 5 resulted in mortality. In contrast, 4/6 strains belonging to clade 2 resulted in mortality. Longitudinal analysis of the disease score indicated that clade 2 strains caused more severe and more prolonged morbidity than the other clinical isolates (Fig. 1D). A chi-squared test of independence revealed that mouse survival is likely dependent on *C. difficile* clade (*P* = 0.012). High acute disease scores correlated with poor survival (*P* < 0.01, based on linear regression).

### Disease severity in mice correlates with disease severity in patients.

**FIG 2:**
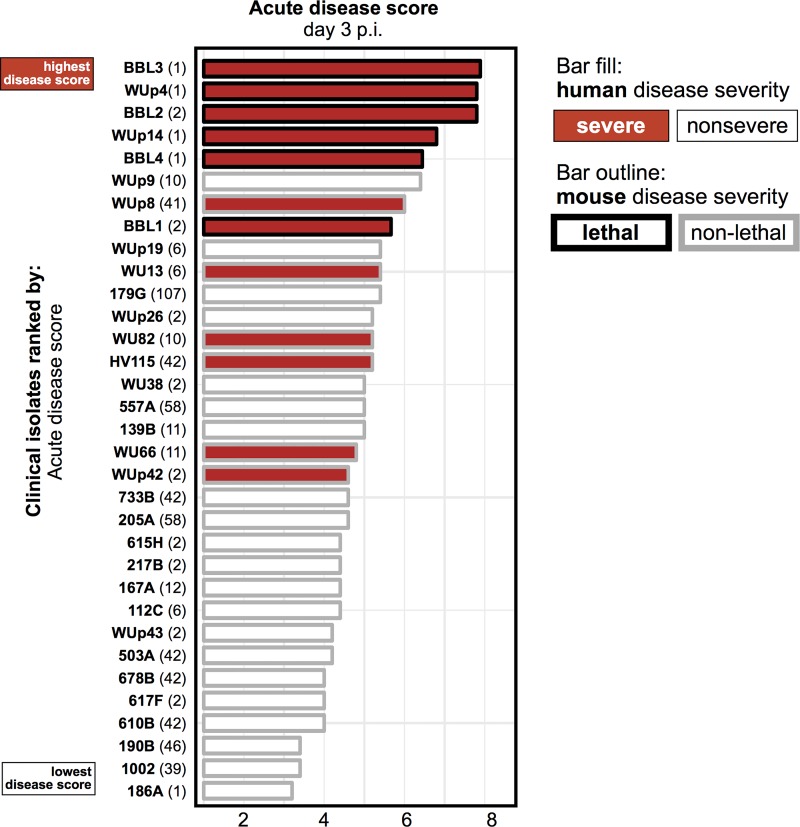
Comparison of *C. difficile* clinical isolate disease severity in humans and mice. Clinical isolates are ranked by their average acute disease score. The bar fill color indicates if the clinical isolate was identified as severe or nonsevere in humans. The bar outlines indicates if the clinical isolate was identified as lethal or nonlethal in mice. Clinical isolates are labeled by their unique strain identifier (shown in boldface) followed by their MLST type (in parentheses).

Use of genetically identical, inbred C57BL/6 mice that were cohoused and treated with the same antibiotic combinations equalized host genetic factors and microbiota composition, thus potentially revealing pathogen-specific variations in virulence. To determine whether disease severity caused by different *C. difficile* isolates was similar in mice and humans, we categorized clinical isolates as “lethal” if they killed at least one mouse in the cohort and “nonlethal” if they did not result in mouse mortality. We then compared the murine disease severity to the severity reported in the infected patients (Fig. 2; Table 1). We found that none of the human nonsevere isolates (0/21) was lethal in mice, whereas 6/12 human severe isolates caused mortality in mice. Although the severity correlation was not perfect, these results suggested that factors that lead to severe disease in humans are also associated with more severe disease and higher mortality in mice.

**TABLE 1:** Classification of *C. difficile* clinical isolates by clade, MLST, and disease severity

Strain	Source	Clade	MLST	Disease severity		Acute disease score
				Human	Mouse	
BBL3	MSK	2	1	Severe	Lethal	6.9
WUp4	WU/BJH	2	1	Severe	Lethal	6.8
BBL2	MSK	1	2	Severe	Lethal	6.8
WUp14	WU/BJH	2	1	Severe	Lethal	5.8
BBL4	MSK	2	1	Severe	Lethal	5.4
WUp9	WU/BJH	1	10	Nonsevere	Nonlethal	5.4
WUp8	WU/BJH	2	41	Severe	Nonlethal	5.0
BBL1	MSK	1	2	Severe	Lethal	4.7
WUp19	WU/BJH	1	6	Nonsevere	Nonlethal	4.4
WU13	WU/BJH	1	6	Severe	Nonlethal	4.4
179G	MSK	1	107	Nonsevere	Nonlethal	4.4
WUp26	WU/BJH	1	2	Nonsevere	Nonlethal	4.2
WU82	WU/BJH	1	10	Severe	Nonlethal	4.2
HV115	MSK	1	42	Severe	Nonlethal	4.2
WU38	WU/BJH	1	2	Nonsevere	Nonlethal	4.0
557A	MSK	1	58	Nonsevere	Nonlethal	4.0
139B	MSK	5	11	Nonsevere	Nonlethal	4.0
WU66	WU/BJH	5	11	Severe	Nonlethal	3.8
WUp42	WU/BJH	1	2	Severe	Nonlethal	3.6
733B	MSK	1	42	Nonsevere	Nonlethal	3.6
205A	MSK	1	58	Nonsevere	Nonlethal	3.6
615H	MSK	1	2	Nonsevere	Nonlethal	3.4
217B^a^	MSK	1	2	Nonsevere	Nonlethal	3.4
167A	MSK	1	12	Nonsevere	Nonlethal	3.4
112C	MSK	1	6	Nonsevere	Nonlethal	3.4
WUp43	WU/BJH	1	2	Nonsevere	Nonlethal	3.2
503A	MSK	1	42	Nonsevere	Nonlethal	3.2
678B	MSK	1	42	Nonsevere	Nonlethal	3.0
617F	MSK	1	2	Nonsevere	Nonlethal	3.0
610B	MSK	1	42	Nonsevere	Nonlethal	3.0
190B	MSK	1	46	Nonsevere	Nonlethal	2.4
1002	MSK	4	39	Nonsevere	Nonlethal	2.4
186A	MSK	2	1	Nonsevere	Nonlethal	2.0

^a^ Clinical isolate 217B was used as the reference strain for pathogenicity locus and core genome analyses.

### *Ex vivo* and *in vitro* experiments explain some, but not all, of the observed differences in acute disease scores.

**FIG 3:**
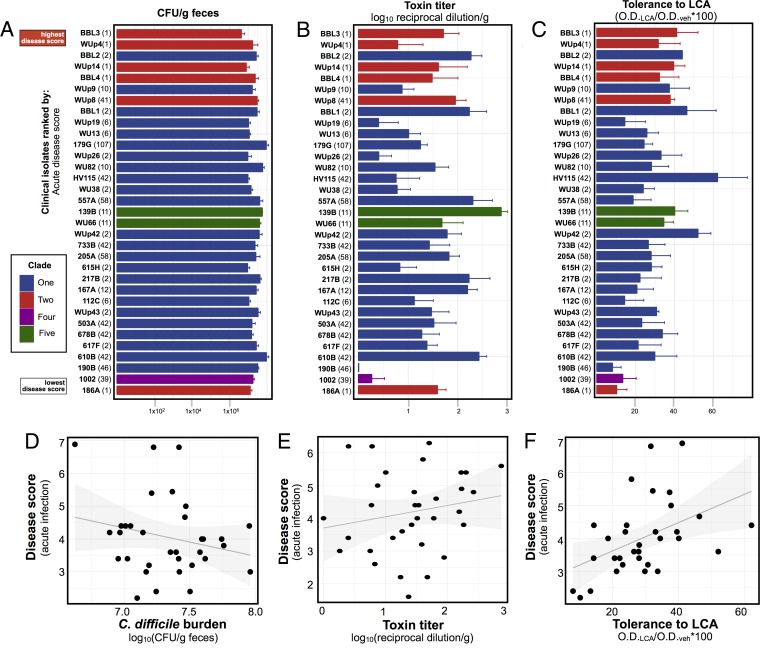
*Ex vivo* and *in vitro* assessments of putative virulence factors of *C. difficile* clinical isolates. (A) *C. difficile* burden in the feces of mice 2 days after infection (*n* = 5 to 10 mice per isolate). (B) Toxin titers in the feces of mice 2 days after infection (*n* = 5 to 10 mice per isolate). (C) Tolerance of each clinical isolate to administration of 0.01% lithocholic acid (*n* = 3 experimental trials per isolate). (A to C) Clinical isolates are ranked by their average acute disease score. They are labeled by their unique strain identifier (in bold) followed by their MLST type (in parentheses). Error bars indicate standard errors. (D to F) Comparison of *ex vivo* and *in vitro* data to disease score, 2 or 3 days postinfection. Solid lines represent the linear regression, with 95% confidence intervals in the corresponding shaded regions.

We hypothesized that the range in disease severity among *C. difficile* clinical isolates could be due to diversity in their ability to colonize mice efficiently. To test this hypothesis, we collected fecal pellets from mice on days 2, 7, and 14 postinfection and quantified the *C. difficile* burden (9). By day 2, all mice were colonized in excess of 4.0 × 10^6^ CFU per gram of stool (Fig. 3A), and they remained colonized for the duration of the experiment (data not shown). The slight variations observed in *C. difficile* burden did not explain the differences in acute disease score (*P* = 0.21) (Fig. 3D).

We next examined each clinical isolate for its production of toxin in an *ex vivo* setting. One commonly cited explanation for the increased virulence of the MLST 1/R027 epidemic strain is that it produces more toxin than preepidemic controls; however, initial studies measured toxin in an *in vitro* setting that may not accurately represent the infectious environment (5, 27). We instead measured toxin titers directly from fecal samples collected from mice 2 days after infection by using a functional, cell-based assay (9). Despite colonizing the mice to similar levels, the clinical isolates produced a wide range of toxin levels during acute infection (Fig. 3B). However, toxin production did not correlate with disease severity (*P* = 0.29) (Fig. 3E). Two other *in vitro* phenotypes that have previously been associated with *C. difficile* virulence, sporulation rate (28, 29) and germination efficiency (30), likewise did not demonstrate an association with acute virulence across our 33 clinical isolates (data not shown).

Finally, recent studies from our lab and others have examined the relationship between secondary bile acid concentrations and the ability of *C. difficile* vegetative cells to grow (10, 14, 31–33). In an *in vitro* setting, the 33 clinical isolates exhibited a broad range of tolerance to the secondary bile acid lithocholic acid (LCA) (Fig. 3C). Isolates from the phylogenetically diverse clade 1 were variable in terms of their ability to grow in the presence of LCA. Of note, high-virulence clade 2 isolates were more tolerant to LCA than the low-virulence clade 2 isolate 186A. Overall, strains with increased lithocholic acid tolerance had higher acute disease scores (*P* = 0.0096) (Fig. 3F). We saw a similar trend with regard to tolerance to the secondary bile acid deoxycholic acid (DCA), but this trend did not reach statistical significance (data not shown).

### Examination of the pathogenicity locus reveals clade-based variability in toxin sequences.

We next used a genome sequence-based approach to identify genomic regions associated with acute virulence scores in the mouse model, beginning with analyses of *C. difficile*’s pathogenicity locus (PaLoc). The PaLoc is a 19.6-kb genetic locus that contains the genes encoding toxins A and B (*tcdA* and *tcdB*, respectively) as well as three small open reading frames (ORFs) that encode putative regulatory elements (*tcdR/tcdD*, *tcdE*, and *tcdC*) (34). Variants of the PaLoc genes have been suggested to account for strain hypervirulence in the human host (27).

**FIG 4:**
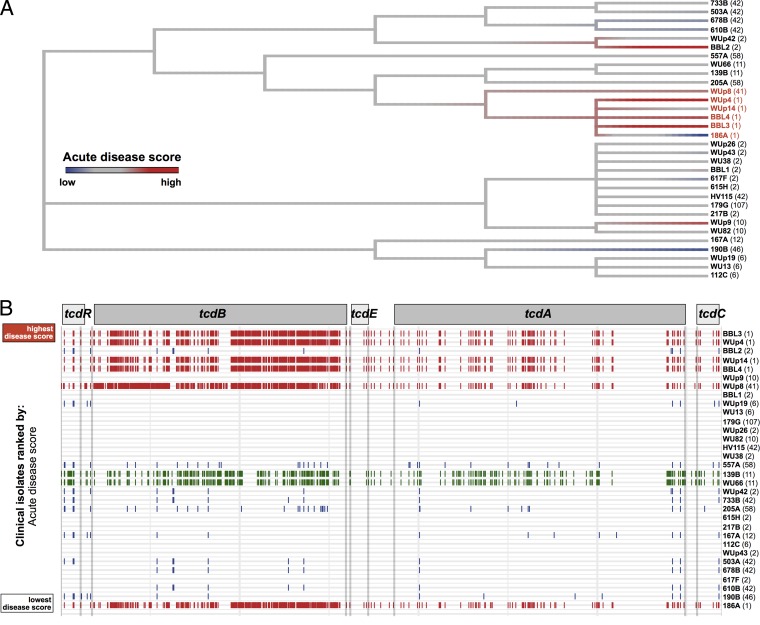
Comparative analysis of *C. difficile* pathogenicity loci. (A) Phylogenetic tree of pathogenicity locus sequences with each clinical isolate. Tree leaves are colored by the relative acute disease score (from blue to red). Clinical isolates are labeled by their unique strain identifier (in bold) followed by the MLST type (in parentheses), and clade 2 isolates are labeled in red text. (B) Single nucleotide variant differences of pathogenicity locus sequences, in relation to the reference strain 217B (MLST 2). Each variant is indicated by a small vertical line, and variants are assigned colors based on the isolate’s clade. Clinical isolates are arranged by their acute disease score.

Using a clade 1, MLST 2 strain (217B) as our reference or comparison strain, we mapped single nucleotide polymorphism variants across the PaLoc and constructed a tree based on these variants (Fig. 4A). We then assigned colors to the tree leaves based on acute disease scores. The tree revealed that the PaLoc sequences clustered based on the clinical isolate’s clade and, to a lesser degree, MLST type. While the high-virulence clade 2 isolates clustered together, other high-virulence strains mapped to more distant branches (see BBL2 and WUp9). Plotting PaLoc variants linearly and arranging each strain based on its acute virulence rank also demonstrated this pattern (Fig. 4B). These two analyses showed that no one variant pattern of the PaLoc is consistently associated with high acute disease scores; high-virulence strains were found to have PaLoc sequences that belonged to at least three distinct sequence patterns (e.g., BBL3, WUp9, and WUp8).

### Examination of the core genome revealed distinct subclusters of high-virulence strains.

Following examination of the pathogenicity locus, we inferred the phylogeny of our clinical isolates by comparing the sequences of their shared, or core, genome. Comparison of all ORFs across the isolates identified the core genome. The gene content within single isolates averaged 3,868 ORFs (range, 3,602 to 4,206 ORFs), and the shared genome was composed of 1,459 ORFs. The sequences of the core genes were then compared in an analysis similar to that of the pathogenicity locus, again using strain 217B as the reference. No two clinical isolates had identical core genome sequences; WUp26 was most closely related to the reference strain, with 87 variants in the core genome.

**FIG 5:**
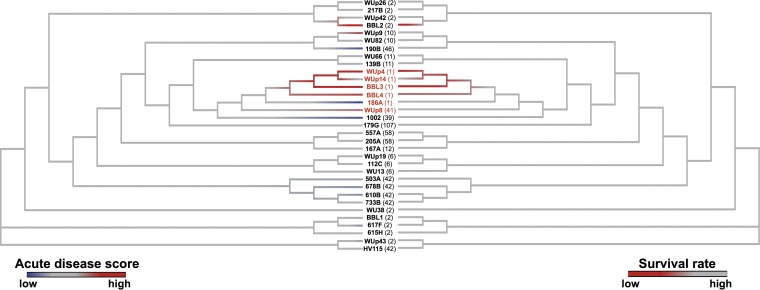
Core genome phylogeny of *C. difficile* clinical isolates. Phylogenetic tree of core genome sequences with each clinical isolate. Tree leaves are colored by acute disease score (left) or survival rate (right). Clinical isolates are labeled by their unique strain identifier (in bold) followed by MLST type (in parentheses), and clade 2 isolates are labeled in red text.

The phylogenetic tree assembled using core gene sequences revealed distinct clustering of the clinical isolates based on their clade (Fig. 5). The isolates also clustered with their MLST group, with MLST 2 and MLST 42 demonstrating the most heterogeneity. Unlike the other MLST groups, MLST 2 and MLST 42 did not fall into discrete lineages. Assigning color to the tree leaves according to acute disease score (Fig. 5, left) or mortality rate (Fig. 5, right) revealed a high-virulence cluster within MLST 1/R027 isolates. In addition, the core genome analysis identified strains whose high virulence was not immediately attributable to their position on the tree (e.g., BBL2, WUp9).

### Analysis of the accessory genome revealed gene groups that were associated with *in vivo* disease scores.

**FIG 6:**
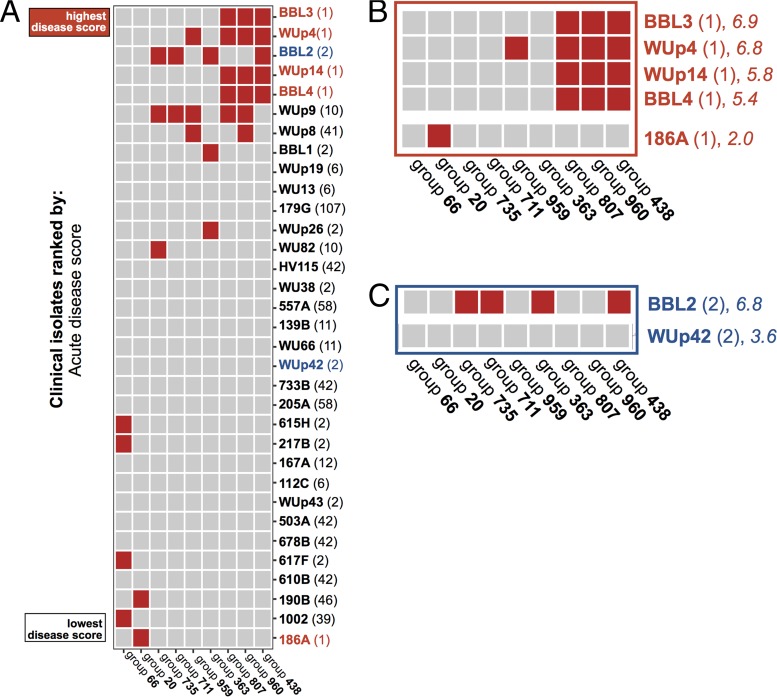
Top discriminant features in the accessory genome, based on multiple regression analysis. (A to C) Presence (red) or absence (gray) of the indicated ORF group for each *C. difficile* clinical isolate. Clinical isolates are labeled by their unique strain identifier (in boldface) followed by the MLST type (in parentheses). (A) Overall multiple regression results. (B) Comparison of low-virulence (186A) and high-virulence MLST 1 strains. (C) Comparison of intermediate-virulence (WUp42) and high-virulence (BBL2) MLST 2 strains.

Excluded from the previous analysis was the accessory genome, that is, the genes not universally shared among the various *C. difficile* isolates. The accessory genome, also known as the flexible or dispensable genome, contains genes nonessential for bacterial growth but often includes factors that provide a given strain with a survival advantage in specific environments (e.g., antibiotic resistance or resistance to detergents) (35). We identified the accessory genome in our *C. difficile* clinical isolates and assembled a large matrix that denoted the presence or absence of each ORF in every strain. This matrix was then simplified by aggregating the ORFs with identical gene presence/absence patterns in all strains. By aggregating the ORFs this way, the accessory genome matrix was reduced in size to 1,156 unique “ORF groups.” Next, we used generalized linear regression to identify which ORF groups were associated with the acute disease score (Fig. 6A).

In contrast to the core genome and pathogenicity locus analyses, which grouped all MLST 1/R027 isolates together despite differences in disease scores (Fig. 4A and 5), examination of the accessory genome demonstrated that the high-virulence MLST 1 strains (BBL3, WUp4, WUp14, and BBL1) all contained ORF groups that were absent from the lone low-virulence MLST 1 strain (186A) (Fig. 6B). These ORF groups were diverse and included transcription-associated proteins, cell surface proteins, and many genes of unknown function (Table 2).

**TABLE 2:** Top discriminant features in the accessory genome^a^

ORF group	ORF(s) identified	Function or annotation	Level of associated virulence
20	8636	Hypothetical protein	Low
	8649	Hypothetical protein	
			
66	1800	Hemolysin XhiA family protein	Low/intermediate
363	1744	DNA-packaging protein	High
438	695	Fis family transcriptional regulator	High
			
711	9089	Hypothetical protein/phage protein	High
	9090	Hypothetical protein	
	9091	Transcriptional regulator	
	9122	Hypothetical protein	
	9123	Hypothetical protein	
	9124	Hypothetical protein	
			
735	9160	Unknown	High
			
807	rep	DNA helicase	High
	recF_1	DNA recombinase	
	3254	Hypothetical protein	
	531	Helicase	
	894	ATPase AAA	
	9165	Single-stranded DNA-binding protein	
	9166	Hypothetical protein	
	9167	Conjugal transfer protein	
	9168	Hypothetical protein	
	9169	Cell surface protein	
	9170	DNA topoisomerase III	
	9171	DNA binding protein	
	9172	Hypothetical protein	
	9173	Transcriptional regulator	
	9175	Transposase	
	9176	Endonuclease	
	9177	Hypothetical protein	
	9178	Conjugal transfer protein	
			
959	14507	Transposase	High
			
960	35	ATP/GTP binding protein	High
	iap_3	Lysozyme-like family protein	

a Each ORF group from the regression analysis was examined for the genes within the group, and the putative functions for each gene are reported.

The accessory genome also helped discriminate between clade 1 isolates with disparate disease scores. This could also be seen with two closely related MLST 2 strains, WUp42 (intermediate virulence) and BBL2 (high virulence). These isolates were highly similar based on pathogenicity locus sequence (Fig. 4A and B) and core genome sequence (Fig. 5), but accessory genome analysis revealed that strain BBL2 contains ORF groups that matched other high-virulence strains irrespective of MLST (Fig. 6C; Table 2).

## DISCUSSION

Although *C. difficile* was first identified as a benign organism colonizing the gastrointestinal tract of infants (36), subsequent investigations identified it as the etiologic agent of pseudomembranous colitis in adults (3). The mechanisms by which *C. difficile* causes severe disease in one individual but silently colonizes another are multifactorial and likely involve host factors, microbiota composition, and *C. difficile* strain differences. To more clearly demonstrate strain-specific differences in *in vivo* virulence, we used a mouse model in which the hosts are genetically identical and the intestinal microbiota and preceding antibiotic treatment were as similar as possible.

Previous work investigating virulence factors in epidemic lineages, such as MLST 1/R027, resulted in conflicting data (37–39). For example, the increased toxin production reported in MLST 1 and MLST 11 isolates was initially attributed to a deletion in the gene *tcdC*, a putative negative regulator of *tcdA* and *tcdB* transcription (27, 40–42). Subsequent studies, however, did not detect increased toxin production when the *tcdC* mutation was introduced into a nonepidemic *C. difficile* strain (43, 44). Along similar lines, *tcdE*, another gene within the pathogenicity locus, is similar to phage holins and was suggested to increase toxin release (45), while others found that toxin release was unaffected when TcdE was functionally inactivated (46). Moreover, the notoriously hypervirulent MLST 1 group was heterogeneous, perhaps explaining the disagreement among published reports (47).

A major hurdle facing previous investigations of *C. difficile* strain type and virulence had been deducing virulence from disease severities in the human host: patients diagnosed with *C. difficile* colitis, in addition to differing in age, underlying disease, and immune status, often received different predisposing antibiotic treatments, chemotherapy, or other medical interventions. Given this heterogeneity, identification of *C. difficile* genotypic correlates to phenotype has been challenging (26). Mice share many of the key aspects of *C. difficile*-associated disease with humans, including antibiotic-induced susceptibility (9, 21, 22), inflammatory cell recruitment (13), and asymptomatic carriage (23). We find that the most virulent *C. difficile* strains in humans are also virulent in mice. However, we also find that some *C. difficile* isolates obtained from patients with severe colitis do not cause severe disease in mice. While this may result from physiological differences between the two host species, it may also result from yet-to-be defined vulnerabilities in some human hosts that are not shared by the mice we have used for our studies.

Of the 33 clinical isolates tested, we found that the majority belonged to *C. difficile* clade 1 and were of relatively low virulence in the mouse model. These strains caused limited disease in mice despite high levels of colonization and intact toxin production, indicating that host innate immune defenses were sufficient to limit mucosal damage. Although mice lacking innate immune defenses can be much more susceptible to *C. difficile* infection (12, 13, 48), it remains unclear whether this applies to all of the less virulent strains we are describing in this report.

In comparison to clade 1 isolates, clade 2 isolates, specifically MLST 1/R027, were more virulent in mice, consistent with the conclusion that MLST 1/R027 strains result in increased mortality in humans (5). However, while it has been suggested that increased virulence of MLST 1/R027 is attributable to increased toxin production, we found that neither the levels of toxin production nor toxin sequence consistently explained differences in *in vivo C. difficile* virulence. Sequence comparison of pathogenicity loci of all clinical isolates revealed that high- and low-virulence MLST 1 isolates shared the same, clade-specific PaLoc variants, indicating that determinants outside the PaLoc contribute to virulence. Thus, neither the levels of toxin production nor toxin sequence explain differences in *in vivo C. difficile* virulence.

Preliminary investigations into *C. difficile* virulence factors found no correlation with either sporulation rates or germination efficiency. It remains possible that these bacterial phenotypes may be involved in *C. difficile* transmission, an avenue not pursued in this study. Tolerance to the secondary bile salt lithocholic acid was the *in vitro* phenotype most strongly associated with *in vivo* disease scores, extending previous work demonstrating that *C. difficile* strains capable of growing in the presence of secondary bile salts are more likely to cause prolonged morbidity (31). In fact, clinical isolates with greater tolerance to the secondary bile acid deoxycholic acid also tended to produce greater morbidity, although in this case the trend was not statistically significant. It has been established elsewhere that *C. difficile* germination and growth are dependent on favorable concentrations of primary and secondary bile acids; antibiotic treatment removes commensal species that generate inhibitory secondary bile acids from germination-promoting primary bile acids and thus facilitates *in vivo C. difficile* growth (33). Transfer of a consortium of commensal bacteria, including one species capable of converting primary bile acids to secondary bile acids, is sufficient to ameliorate *C. difficile*-induced morbidity in mice (10). However, there is limited data examining the variability of secondary bile acid sensitivity across different *C. difficile* strain types. We hypothesize that secondary bile acid tolerance enhances growth of *C. difficile* in the lower intestine, leading to more prolonged and severe colitis. The precise mechanism by which lithocholic acid tolerance by *C. difficile* increases *in vivo* disease severity remains to be defined.

While many studies have focused on established *C. difficile* virulence determinants, *ex vivo* and *in vitro* experiments combined with whole-genome sequencing of phenotypically distinct *C. difficile* isolates can provide a genome-wide perspective on virulence and pathogenicity. We therefore sequenced the genomes and annotated each of the 33 *C. difficile* strains, and we identified the core genome (shared by all strains in the data set) and the accessory genome (present in only a subset of the strains). As expected, clustering by core genome similarity grouped clinical isolates according to their MLST type, although MLST 2 and MLST 42 strains demonstrated some heterogeneity. Although the low-virulence MLST 1 isolate (186A) clustered separately from the high-virulence MLST 1 isolates, its core genome was more closely related to clade 2 than the other clinical isolates.

Accessory genome analyses identified genomic differences associated with acute disease score. Specifically, the low-virulence MLST 1 strain 186A was found to be missing a number of genes, such as genes for DNA-associated proteins (*rep*, *recF*) and for a membrane proteins (*iap*), that were present in high-virulence MLST 1 and clade 1 strains. The functions of many of these genes and their possible association with *in vivo* virulence remain to be explored. While our study demonstrates that *in vivo* virulence of *C. difficile* can be influenced by genes outside the PaLoc, it remains unclear whether the products of these genes represent potential targets for therapeutic intervention. Further studies that precisely define the contributions of non-PaLoc genes to virulence may lead to diagnostic tests that can distinguish between high- and low-virulence *C. difficile* strains in the clinical setting and to novel therapies that reduce intestinal damage and inflammation.

## MATERIALS AND METHODS

### *C. difficile* clinical isolate collection and classification.

Fecal samples were collected from patients previously identified as colonized or infected with *C. difficile* and plated anaerobically onto brain heart infusion (BHI) agar plates supplemented with yeast extract, cysteine, and the antibiotics cycloserine and cefoxitin (BHI and yeast extract were from BD Biosciences, and the other components were from Sigma-Aldrich). Individual colonies that were able to grow in the presence of these antibiotics and that had the characteristic phenotype of *C. difficile* were selected, isolated, and then typed using MLST (24).

### Mouse husbandry.

All experiments were performed with wild-type female C57BL/6 mice, aged 6 to 8 weeks, purchased from the Jackson Laboratories. Mice were housed in the specific-pathogen-free facility at Memorial Sloan Kettering’s Animal Resource Center, fed irradiated feed, and provided with acidified water. Two of us (B.B.L. and R.A.C.) performed all mouse experiments, including replenishing food as needed and changing cages at least once per week. The experiments were performed in compliance with Memorial Sloan Kettering’s institutional guidelines and were approved by its Institutional Animal Care and Use Committee.

### *In vivo* (murine) virulence assessment of clinical isolates.

*C. difficile* spores for each clinical isolate were prepared as previously described (49). Briefly, clinical isolates were grown anaerobically in liquid BHI medium until the majority of bacterial cells visualized under a light microscope were spores, i.e., approximately 1 to 2 weeks. Spores were purified using a density gradient as previously described and enumerated by plating serial dilutions of the spore stock onto BHI agar plates supplemented with yeast extract, cysteine, and 0.01% taurocholic acid to produce germination (49). Mice were rendered susceptible to infection by pretreating them with a cocktail of antibiotics, metronidazole (0.25 g liter^−1^; Sigma-Aldrich), vancomycin (0.25 g liter^−1^; Nova-Plus), and neomycin (0.25 g liter^−1^; Sigma-Aldrich), which was added to the drinking water for 3 days, after which it was replaced with untreated water. Two days after antibiotic cessation, mice were injected intraperitoneally with 200 µg clindamycin (Sigma-Aldrich). The next day, (designated day 0), the mice were each infected with 100 spores of a given *C. difficile* clinical isolate via oral gavage. Mice were housed in groups of five, and the cages were mixed prior to *C. difficile* infection to help control for intercage variability in microbiota composition (all mice within each cage were infected with the same strain). Each clinical isolate was tested in 5 to 10 mice.

Mice were monitored every day for the first week and every 3 to 4 days during the second week after infection for measures of disease severity as described previously (12). Briefly, measures of weight loss, surface body temperature, diarrhea severity, and phenotypic morbidity were scored and combined to produce a total disease score. In addition, on days 1, 2, 7, and 14 postinfection, fecal pellets were collected for quantification of *C. difficile* burden (9).

### Quantitative *C. difficile* culture and toxin A and B titer determinations.

Bacterial burden and toxin titers in fecal samples on day 2 postinfection were assessed as previously described (9).

### *In vitro* assessment of sporulation rates and germination efficiency.

The sporulation rate of each clinical isolate was determined using previously published protocols (50). Each experiment was repeated twice, for a total of three trials. Germination rates in the presence and absence of taurocholic acid were assessed as previously described (51).

### *In vitro* assessment of secondary bile acid tolerance.

The effects of lithocholic acid and deoxycholic acid on *C. difficile* clinical isolate growth were measured in an *in vitro* setting as previously described (31). Briefly, pure cultures of each isolate were incubated anaerobically at 37°C, and growth was monitored by measurement of the optical density at 600 nm (OD_600_) over a 22-h period. Each isolate was grown under two treatment conditions: (i) vehicle (brain heart infusion medium supplemented with yeast extract and cysteine) or (ii) vehicle with 0.01% lithocholic acid or 0.01% deoxycholic acid. We repeated each experiment twice, for a total of three trials. Tolerance to LCA or DCA treatment was calculated at the exponential phase of culture growth.

### Whole-genome sequencing, assembly, and annotation of clinical isolates.

Single clones of *C. difficile* clinical isolates were grown anaerobically overnight at 37°C in brain heart infusion medium supplemented with yeast extract and cysteine. DNA was extracted using phenol-chloroform with bead beating and purified with a Qiagen QiaAmp kit. Purified DNA was sheared using a Covaris ultrasonicator and prepared for Illumina sequencing with a Kapa library preparation kit with Illumina TruSeq adaptors to create 300- by 300-bp nonoverlapping paired-end reads (10).

Quality control of raw sequence reads was performed using Trimmomatic version 0.35 (52). Trimmed reads were assembled into contigs using the short read assembler SPAdes (v.3.6.1) (53), and secondary scaffolding was performed with AlignGraph using published *C. difficile* reference sequences to guide the assembly (54). Quality assessment of finished assemblies was performed using Quast (v.4.4) (55). The assemblies were then annotated with putative open reading frames using Prokka (v.1.12) (56), and the core, accessory, and pan-genomes were identified with Roary (v.3.7.0) (57). Briefly, genes that were identified in all clinical isolates were analyzed as the core genome, and genes that were present in a subset of the clinical isolates were analyzed as part of the accessory genome. The core genome alignment was performed using Parsnp (v.1.2; Harvest suite) (58) using strain 217B as the reference.

### Pathogenicity locus analysis.

The pathogenicity locus was identified in the clinical isolates using Prokka annotations (56) as well as BLAST alignments (59). The PaLoc alignment was calculated using Parsnp (Harvest suite) (58) using strain 217B as the reference.

### Comparing phylogeny to phenotype outcomes.

The phylogenetic trees calculated by Parsnp for the pathogenicity locus variants and core genome variants were mapped to phenotypic outcomes (acute disease score, survival) by using the phytools (60) and ape (61) packages in R (version 3.3.2) (62). Phylogenetic signal between phenotypic outcomes and the phylogeny was assessed using the phylosignal (63) package in R, and Moran’s *I* index was noted. Because *P* values of >0.05 were used for both the pathogenicity locus and core genome determination, we could not reject the null hypotheses for any of the phylogeny-trait pairs, and we did not perform any phylogenetic signal corrections.

### Accessory genome analysis.

The accessory genome was identified with Roary, based on sequence annotations provided by Prokka. The accessory genome was compiled into a matrix that defined the presence (score of 1) or absence (0) of each open reading frame in all clinical isolates. This matrix was then consolidated so that ORFs with the same presence/absence pattern in each isolate were aggregated into “ORF groups.” The glmnet package (64) in R was then used to fit a generalized linear model to determine which ORF groups were able to predict the acute disease scores of the clinical isolates.

### Statistical analysis.

A chi-square test of independence was run to test if mouse survival was likely dependent on *C. difficile* clade. The test was run twice: comparing clade 2 survival against all non-clade 2 strains and for clade 2 versus clade 1 alone. Linear regression was used to assess if acute disease score was associated with survival rates in mice. Linear regression was also used to compare if the *ex vivo* and *in vitro* phenotypes were associated with acute disease scores. Statistical significance was determined by using a cutoff *P* value of <0.05.
